# Editorial: Pitfalls in the Neuro-Imaging of Glioblastoma in the Era of Antiangiogenic and Immune-Targeted Therapy—Detecting Elusive Disease, Defining Response

**DOI:** 10.3389/fneur.2017.00311

**Published:** 2017-07-03

**Authors:** Pamela Zyman New, Myrna Rachel Rosenfeld

**Affiliations:** ^1^Houston Methodist Research Institute, Houston, TX, United States; ^2^Institute for Biomedical Investigations – Hospital Clinic, Barcelona, Spain

**Keywords:** glioma, positron-emission tomography, magnetic resonance imaging, EEG, CT scan

**Editorial on the Research Topic**

**Pitfalls in the Neuro-Imaging of Glioblastoma in the Era of Antiangiogenic and Immune-Targeted Therapy—Detecting Elusive Disease, Defining Response**

High grade glioma remains a lethal disease and a treatment challenge. Current standard of care includes maximum safe surgical resection, followed by radiotherapy with concurrent chemotherapy then adjuvant chemotherapy, resulting in average survival of 14–15 months. The history of research in glioblastoma has evolved. In the 1970s, new agents were not evaluated in glioma until their early phase testing in systemic cancer had been completed, resulting in years of delay. Determining valid endpoints and response criteria also developed slowly, as responses in glioblastoma were not common early in the field. Prior to the existence of MRI, Dr. Victor Levin (1977) (1) developed a method of response assessment which included nuclear medicine brain imaging, CT scan, EEG, and neurological evaluation. In 1981, the World Health Organization (WHO) reported the results of two international meetings called to establish a standardization of response assessment and reporting of results as investigational treatments moved forward in medical oncology (2). Objective responses could be determined clinically, radiographically, biochemically, or by surgical pathological restaging. Bi-dimensional measurements were used and verification of response was required after 4 weeks.

As MRI became increasingly available, it became the imaging technique of choice in neuro-oncology. Today, it remains a very important method to assess response or disease progression and is used in the decision-making plan to continue or discontinue specific therapies for individual patients and in the construct of clinical trials. However, the use of standard MRI is being challenged by the development of new agents which affect the integrity of the blood brain barrier, the immune system, and the molecular structure of cancer cells. The unique effects of these agents require revisiting how MRI is used in assessing response as well as the consideration of applying new imaging technologies. Because of these concerns and recent issues in determining treatment progression versus pseudo-progression in the context of these new therapies, this project was launched to obtain opinions, background, research, and views to the future from those on the cutting edge of the application of neuro-imaging in Neuro-oncology. Part I of this endeavor is collected here.

The topics and imaging modalities discussed in this collection include the status of MRI imaging and different sequences which can be useful, PET scanning with various amino acid tracers, and the application of mathematical functions to perfusion MRI which can improve the accuracy of interpretation. Dr. Huang et al. starts off the series with an excellent review of the development of imaging criteria for response, beginning with the WHO criteria, the MacDonald criteria that incorporated the bi-dimensional measurement of enhancing tumor with corticosteroid dose and neurological status, and finally response assessment in neuro-oncology, which takes into account MRI T-2 weighted and FLAIR images, an important addition for assessment of invasive disease and new treatment modalities. Dr. Huang et al. discusses various imaging strategies including perfusion MRI, MR spectroscopy, new PET tracers, as well as the importance of algorithms and interpretation methods. He describes the complexities of the three perfusion techniques, dynamic susceptibility contrast (DSC) MRI, dynamic contrast enhanced MRI, and arterial spin latency, from the standpoint of implementation and interpretation. Attributes and limitations of diffusion tensor imaging are also discussed. Dr. Huang et al. reviews the challenge of imaging patients treated with anti-angiogenic agents as well as the need for guidelines in the interpretation of imaging in patients receiving the immune therapies. He emphasizes the need to improve accuracy and reliability as well as the need for validation of promising new techniques.

Dr. Steven Chiang discusses PET molecular imaging, which is founded on the use of a radioactive substrate that is metabolized by tumors; thus, FDG is taken into the intracellular space of a neoplasm as would be glucose. He reviews the mechanism by which cancer cells concentrate the radioactive substrate, that can also be taken up by normal brain cells and inflammatory cells, resulting in false positive data. He also discusses the usefulness of serial imaging and the view that multiple imaging modalities may be important for the accurate assessment of disease recurrence and treatment effect. He concludes with a look to the newer agents in molecular imaging as added benefit.

Drs. Norbert Galldiks and Karl-Josef Langen present the attributes of amino acid PET in the early detection of tumor recurrence and share their evidence for improved differentiation of tumor recurrence versus therapeutic effect. They review issues of blood–brain barrier breakdown not related to tumor progression. They also discuss the difficulty in imaging patients who are receiving anti-angiogenic therapy and problems in relying on T-2 MRI and FLAIR sequences alone. Drs. Norbert Galldiks and Karl-Josef Langen discuss the usefulness of amino acid PET tracers after radiotherapy, brachytherapy, and anti-angiogenic treatment. They also address the issue of radiation necrosis especially following stereotactic radiosurgery and present the concept of tumor to brain ratio measurements.

Finally, Dr. Wong et al. further explores the technique of DSC perfusion imaging as it is used in the diagnosis of malignant brain tumors. He addresses the question of how to differentiate true tumor progression versus pseudo-progression with the aid of mathematical algorithms, especially as they apply to regional cerebral blood flow (rCBF) and cerebral blood volume. His paper discusses the need for standardization of post-processing algorithms in the application of DSC perfusion. Dr. Wong et al. points out areas of potential error in measurement of rCBF using deconvolution methods and tissue residue function. He discusses the effect of signal truncation in DSC perfusion studies of glioblastoma patients in the context of gene-mediated cytotoxic therapy.

In summary, part I of this research topic addresses issues of MR and PET imaging that are crucial to response assessment and the challenges presented by the applied imaging techniques as well as the complex molecular biology of this devastating tumor. A theme that arises from these excellent reviews is that one imaging methodology may not be sufficient to determine progression of disease versus pseudo-progression, and as we continue to explore and validate imaging techniques that improve sensitivity and specificity, a combination of modalities may be the solution.

## Author Contributions

All authors listed have made a substantial, direct and intellectual contribution to the work, and approved it for publication.

## Conflict of Interest Statement

The authors declare that the research was conducted in the absence of any commercial or financial relationships that could be construed as a potential conflict of interest.
